# The second victim phenomenon among German emergency medical technicians: a cross-sectional study based on the SeViD questionnaire (SeViD-VIII)

**DOI:** 10.1186/s12873-025-01298-6

**Published:** 2025-07-28

**Authors:** Hartwig Marung, Victoria Klemm, Reinhard Strametz, Thomas Neusius, Matthias Raspe, Hannah Roesner, Harald Karutz, Klaus Runggaldier, Rainer Petzina, Luis Teichmann, Stefan Bushuven

**Affiliations:** 1 Institute for Safety of Patients and Health Professionals (ISPP), MSH Medical School Hamburg, Am Kaiserkai 1, 20457 Hamburg, Germany; 2 Wiesbaden Institute for Healthcare Economics and Patient Safety (WiHelP), RheinMain University of Applied Sciences, Bleichstraße 44, 65183 Wiesbaden, Germany; 3 Department of Internal Medicine, Infectious Diseases and Respiratory Medicine, Charité– Universitätsmedizin Berlin, Corporate Member of Freie Universität Berlin, Humboldt-Universität zu Berlin, Berlin Institute of Health, Charitéplatz 1, 10117 Berlin, Germany; 4 Rheinisch-Westfälische Technische Hochschule RWTH Aachen, Faculty of Medicine, Pauwelstrasse 30, 52074 Aachen, Germany; 5 Training Center for Emergency Medicine (NOTIS e.V.), Vilstalstraße 26A, 87459 Pfronten, Germany; 6 Department of Anesthesiology and Critical Care, University of Freiburg Medical Center, Freiburg, Germany

**Keywords:** Second victim, Emotional distress, Paramedics, Emergency medical technicians, Psychological distress, Patient safety, Occupational health

## Abstract

**Background:**

Emergency medical technicians (EMTs) frequently encounter high-stress, traumatic events, making them vulnerable to the second victim phenomenon (SVP), a state of emotional distress following adverse patient-related incidents. While SVP is well documented among physicians and nurses, research on EMTs remains limited. This study examines the prevalence, risk factors, symptom burden, and preferred support strategies for SVP among German EMTs.

**Methods:**

A cross-sectional survey was conducted using the validated SeViD questionnaire (Second Victims in German-speaking countries). The survey assessed SVP prevalence, symptom severity, and preferred support measures. Binary logistic regression was performed to identify predictors of SVP and symptom burden. Descriptive statistics were used to summarize demographic and occupational characteristics.

**Results:**

Among the 699 respondents, 528 (75.5%) completed the survey. The prevalence of SVP was 65.3%, with 53.3% reporting SVP within the past 12 months. The most common triggering events were unexpected patient deaths (37.1%) and aggressive behavior from patients or relatives (19.1%). Logistic regression revealed that professional experience (OR = 1.055, *p* < 0.001) and employment in ground-based intensive care transport (OR = 2.444, *p* = 0.004) were risk factors for SVP, whereas male gender (OR = 0.392, *p* < 0.001) and conscientiousness (OR = 0.765, *p* = 0.033) were factors associated with lower risk. Higher extraversion was associated with lower symptom burden (OR = 0.754, *p* = 0.013). The most valued support measures were legal consultation and professional counseling.

**Conclusions:**

SVP is highly prevalent among EMTs and has significant psychological and emotional consequences. Greater work experience and intensive care transport roles increase SVP risk, whereas conscientiousness and extraversion appear protective. The implementation of structured peer support programs may help mitigate the impact of SVP.

## Background

Emergency medical technicians (EMTs) are pivotal in providing immediate medical assistance in prehospital settings and respond swiftly to emergencies worldwide. As frontline responders, EMTs are often the first to arrive at scenes of traumatic incidents, exposing them to a multitude of distressing and high-stakes situations affecting patient safety [1]. These situations include not only critical medical situations in infants, children, and adults (such as cardiac arrest, shock, respiratory failure, trauma, and suicide) but also mass casualty incidents (MCIs) and the experience of violence against themselves or cooperating forces such as police and firemen. Furthermore, the overcrowding of emergency departments makes the admission of patients (especially frequent users) difficult and even conflictful. Recent staff shortages among EMTs contribute to extra hours, shorten recovery times, and increase uncertainties in their personal life and families. Despite their fundamental role in emergency care, EMTs often operate in environments where feedback regarding patient outcomes is limited, negatively influencing overall job satisfaction [2]. Furthermore, the lack of general routine debriefing sessions within emergency medical response systems may leave EMTs without structured opportunities to process and address the emotional toll of their experiences. This holds true for most of the paramedics from the German EMS system and has been described for EMS systems in other regions of the world, e.g. Saudi-Arabia, while recent research emphasizes the positive impact of debriefing on EMS personnels` psychological wellbeing [3–5]. This combination of factors underscores the unique challenges that EMTs face and the heightened risk of experiencing the Second Victim Phenomenon (SVP) within this population.

Second victims status is defined as “any health care worker, directly or indirectly involved in an unanticipated adverse patient event, unintentional healthcare error, or patient injury, and who becomes victimized in the sense that they are also negatively impacted” [6]. SVP is often characterized by emotional distress and psychological trauma experienced by healthcare professionals following adverse patient events, and is an increasingly recognized concern across various healthcare settings [7, 8]. While previous research has focused primarily on physicians [9–11] and nurses [12, 13], there is a growing recognition of the need to extend investigations into other frontline healthcare roles, including EMTs. The SeViD-III study investigated the SVP among German emergency physicians and self-reported a prevalence of 53% [10]. Emergency physicians working outside hospitals in the field are limited to some countries, including Germany. Other nations use paramedic-based systems. Concerning the high prevalence of SVP among these physicians, the same situation among EMTs is likely but underinvestigated. The SeViD-VIII study aims to fill this gap in research by investigating the prevalence, characteristics, and associated factors of SVP among EMTs. Addressing the prevalence and impact of SVP among EMTs is essential for promoting their well-being and enhancing patient, provider, institutional and system safety.

## Methods

### Design and conduction of the SeViD-VIII survey

This study is a cross-sectional study conducted among EMTs. As we had no indication of regional differences from previous SeViD studies, questionnaires were sent to the entire German prehospital emergency system. Inclusion criteria were certified EMS technicians or paramedics from the German EMS system and informed consent to participate. Exclusion criteria were insufficient knowledge of the German language and incomplete questionnaires. Data collection via convenience sampling was anonymized completely with neither tokens, cookies nor IP addresses stored. We used the SurveyMonkey platform (San Mateo, CA, USA) to conduct the study. Invitations were sent via a professional network of major German EMS organizations. The Strengthening the Reporting of Observational Studies in Epidemiology (STROBE) checklist for cross-sectional studies was used to guide the reporting of the results [14].

The study was conducted over a four-week period from July 8 to August 5, 2023. Reminders were sent out at the two-week and three-week marks. Invitations and reminders included statements about the survey’s purpose, details about the responsible researchers (with a contact email for questions), information on the study’s anonymity, length, voluntary participation, and data protection. The survey could only be accessed via a provided link or QR code. Owing to complete anonymization, it was not possible to control for multiple participants or the distribution of the invitation link outside the target population.

Ethical review and approval were waived for this study after the concept was presented to the ethical committee of the medical association of Hesse, Germany.

### Construction and validation of the SeViD-questionnaire

The comprehensive construction and validation of the questionnaire are detailed in another publication [15]. The questionnaire was developed in German via six existing questionnaires identified in a systematic literature search. It consists of three domains and 40 items. Content validity was ensured via cognitive pretesting by an independent researcher. Table 1 shows the structure of the final questionnaire.

**Table 1:** The validated SeViD questionnaire

Domain	Item
General experience with the SVP	Knowledge of the term
	Lifetime prevalence
	12-month prevalence
	Key incident
	Seeking support after the incident
	Groups offering support after the incident
	Self-perceived time of recovery
Second Victim symptoms	Fear of exclusion by colleagues
	Fear of losing the job
	Listlessness
	Depressive mood
	Concentration difficulties
	Reliving the situation outside of professional life
	Reliving the situation in similar professional situations
	Aggressive, risky behavior
	Defensive, overly cautious behavior
	Psychosomatic reactions (head- or backaches)
	Insomnia or excessive need for sleep
	Use of alcohol/drugs because of event
	Feelings of shame
	Feelings of guilt
	Self-doubts
	Social isolation
	Anger towards others
	Anger towards myself
	Desire for support from others
	Desire to process the event for better understanding
Support measures	The possibility to take time off from work directly to process the event
	Access to professional counseling or psychological/psychiatric consultations (crisis intervention)
	The possibility to discuss my emotional/ethical thoughts
	Clear and timely information regarding the course of action after a serious event (e.g., damage analysis, error report)
	Formal emotional support in the sense of organized collegial help
	Informal emotional support
	Quick processing of the situation/quick crisis intervention (in a team or individually)
	Support/Mentoring when continuing to work with patients
	Support when communicating with patients and/or relatives
	Guidelines regarding the role/activities expected of me during a serious event
	Support to be able to take an active role in the processing of the event
	A secure possibility to give information on how to prevent similar events in the future
	The possibility to access legal consultation after a severe event

The symptoms were rated on 3-point Likert-scales (1 = not pronounced, 2 = weakly pronounced, 3 = strongly pronounced), and the support measures were rated on 4-point Likert-scales (1 = not helpful at all; 2 = not very helpful; 3 = somewhat helpful; 4 = very helpful). In order to avoid a bias by explaining the term “Second Victim” beforehand, participants were asked if the definition of the term had been known to them before starting the survey, with “yes” or “no” as answering options. Answering this question was mandatory before moving on to the next question, where the definition of the SVP was provided. The survey employed adaptive questioning, meaning that questions in the symptom domain were shown only to participants who had experienced second victim incidents. The participants could leave comments before completing the survey.

In addition to the SeViD-questionnaire, nine items were included to assess baseline characteristics, such as age, gender, work experience, workplace, qualifications, and characteristics of the workplace (number of people covered by the EMS, type of area covered by the EMS, working mode, and work time). We have also added the question of whether the event leading to SVP was linked to the SARS-CoV-2 pandemic. This study is the eighth out of a series of ten SeViD studies. That series was started during the pandemic, and we decided to leave the specific question within the validated questionnaire. Furthermore, the survey included the validated Big Five Inventory (BFI-10), which measures personality structure across five dimensions: openness, conscientiousness, extraversion, agreeableness, and neuroticism (two items per dimension on a 5-point scale from “strongly disagree” to “strongly agree”) [16].

### Statistical analysis

Demographic variables and applied instruments were summarized through descriptive statistics, with means (M) and standard deviations (SD) presented for interval-scaled data, whereas numbers (n) and percentages (%) were used for nominal and ordinal scaled variables. The analysis was conducted via SPSS Statistics Version 29 (IBM, New York, NY, USA) and the python module stats models [17]. Significance was defined as a p-value less than 0.05. As an explorative study to generate new hypotheses, we did not adjust for multiplicity.

Two exploratory binary logistic regression analyses were carried out. One to identify possible risk factors for becoming a SV and one to identify risk factors associated with a higher or lower symptom load.

To perform the first binary logistic regression analysis, we dichotomized the variable “Lifetime prevalence”, which asked if the participants had experienced SVP. To achieve this, we summarized the answer options “yes, once” and “yes, more than once” into one category and left the answer “no” as the other category. The independent variables we planned to include in the model were gender, age, work experience, work area and the BFI-10 personality traits of neuroticism, openness, conscientiousness, agreeableness, and extraversion.

To perform the second binary logistic regression analysis, we performed a median split to dichotomize the overall symptom load into two categories 0 = low symptom load and 1 = high symptom load. To do so, we first calculated an overall symptom load sum score. We then performed the median split to obtain the two outcome categories. We planned to include gender, age, work experience, type of adverse event leading to SVP, receiving support (yes vs. no), and BFI-10 personality traits as independent variables.

Before performing both binary logistic regression analyses, we checked whether the model met the necessary assumptions. These assumptions included a sufficient sample size of at least ten participants in both categories of the binary dependent variables per independent variable, the linearity of log odds for continuous variables, and no presence of multicollinearity. For binary logistic regression, it is recommended that the model includes only one independent variable per 10 participants in both binary dependent variables [18]. In our model investigating factors influencing the occurrence of SVP, we had 345 participants that identified as SVs and 183 that did not. Therefore, we could only include a maximum of 18 independent variables in our model. We have included 14 and therefore met the above-mentioned recommendations by Backhaus et al. There was a sufficient sample size to include all planned independent variables in the model. The assumption of linearity of log odds was tested, and no violations were found. To detect signs of multicollinearity, we inspected the correlation matrix as well as the variance-inflation factor (VIF). For the correlation matrix, values above 0.7 and VIF values above 5 were considered signs of multicollinearity [19]. Signs of multicollinearity were present for the variables “age” and “work experience”. We therefore excluded “age” from both analyses.

To analyze both models’ fits, explanatory powers, and abilities to discriminate between the possible binary outcomes of the chosen predictors, we calculated Nagelkerke’s pseudo-R² and the receiver operating characteristic (ROC) curve to inspect the area under the curve (AUC) [20].

Since another aim of the study was to detect differences in ratings of possible support measures between SVs and non-SVs, we performed the Mann‒Whitney U test for independent samples. To measure the effect size of the significant differences between the two groups, we calculated Pearson’s correlation coefficient (r) via the equation $\:\varvec{r}=\frac{\varvec{z}}{\sqrt{\varvec{N}}}$ [20].

Before the analyses, the data were checked for missing values. We excluded cases with a greater number (at least five answers to central questions missing consecutively) of systemically missing values (171 in total) [21, 22]. Singular missing data were handled by listwise exclusion of the cases for the analyses [18].

## Results

### Descriptive baseline analysis

A total of 699 EMTs responded, and 528 completed the survey and were therefore included in the analysis (completion rate 75.5%). The mean duration for completion of the survey was 8 min 24 s.

The mean age of the participants was 31.7 years (median (md) = 29; standard deviation (SD) = 10.48; min = 18; max = 65). Most of the participants were male (60.2%, *n* = 318), 39.4% (*n* = 208) were female and 2 did not specify their gender (0.4%). The work experience ranged from one to 40 years with a mean work experience of 9.0 years (SD = 9.04).

Most of the participants did not work in a leading position (84.7%, *n* = 447) and worked full-time (82.6%, *n* = 436). They were given a list of possible work areas for EMTs and were asked to indicate where they mainly categorized themselves (multiple answers were possible). Almost all worked in ground-based rescue (94.9%, *n* = 501), followed by qualified patient transportation (46.8%, *n* = 247). The lowest percentage of participants worked in air-bound intensive care transportation (2.1%, *n* = 11). Most worked in shifts including night shifts (74.2%, *n* = 392). The participants were also asked to characterize the area covered by their EMS (emergency medical service) as shown in Table 2. For Germany, it is generally accepted that EMS systems serving communities with a population of more than 100.000 are considered “urban”.

**Table 2:** Characteristics of the EMS-covered areas

Characteristics		Percentage (%) of Participants (*n*)
Number of people covered by EMS	< 100,000	19.7 (104)
	100,000-250,000	39.6 (209)
	250,000-500,000	26.9 (142)
	500,000–1,000,000	7.0 (37)
	> 1,000,000	6.8 (36)
Type of area covered by EMS	Mixed rural and urban	39.2 (207)
	Rural	38.8 (205)
	Urban	22.0 (116)

When asked about SVP, most participants stated that they did not know the term (74.4%, *n* = 393), although after explaining it 65.3% (*n* = 345) self-identified as SVs. Among those 65.3% of the respondents, more than half stated that they had experienced situations leading to SVP more than once (54.8%, *n* = 189). Furthermore, over half have become SVs within the past twelve months (53.3%, *n* = 184). The most important key-event leading to SVP was the unexpected death or suicide of a patient (37.1%, *n* = 128), followed by the aggressive behavior of patients or their relatives (19.1%, *n* = 66). A total of 12.2% (*n* = 42) of SVP experiences were triggered by other events not listed in the questionnaire and explained further in free text:

“20-year-old traffic accident victim”

“3 children from my neighborhood died in a traffic accident”

“at a crime scene with multiple deaths, one being a child”

“unsuccessful resuscitation of a 13-year-old”

“extended suicide involving two children”

“Pediatric resuscitation” (stated multiple times)

Most of the 345 self-identified SVs received help from others (67.5%, *n* = 233). Of those who did not receive help, 71.1% (*n* = 83) did not ask for help, whereas 29 persons asked but did not receive help. Most support for SVs came from colleagues (89.7%, *n* = 209), followed by family/friends (57.5%, *n* = 135) and counsellors/psychotherapists/psychologic counseling (39.9%, *n* = 93). Support from supervisors (34.8%, *n* = 81) and management (5.2%, *n* = 12) played only a minor role for the participants of this survey. Most participants who had experienced SVP themselves had recovered from it within a month (69.3%, *n* = 239). Fifty participants reported not yet having recovered (14.5%).

Table 3 shows the pronunciation of the symptoms the SVs experienced.

**Table 3:** Symptoms of the SVs included in this survey

Symptom	Not at all (*n*)	Weakly pronounced (*n*)	Strongly pronounced (*n*)
Fear of exclusion by colleagues (*n* = 332)	69.0% (229)	21.4% (71)	9.6% (32)
Fear of losing the job (*n* = 341)	80.6% (275)	13.8% (47)	5.6% (19)
Listlessness (*n* = 339)	42.5% (144)	38.0% (129)	19.5% (66)
Depressive mood (*n* = 340)	25.3% (86)	51.5% (175)	23.2% (79)
Concentration difficulties (*n* = 338)	31.4% (106)	46.1% (156)	22.5% (76)
Reliving the situation outside of professional life (*n* = 330)	33.3% (110)	43.4% (143)	23.3% (77)
Reliving the situation in similar professional situations (*n* = 335)	25.4% (85)	44.5% (149)	30.1% (101)
Aggressive, risky behavior (*n* = 332)	80.1% (266)	15.1% (50)	4.8% (16)
Defensive, overly cautious behavior (*n* = 333)	44.7% (149)	38.4% (128)	16.9% (56)
Psychosomatic reactions (head- or backaches) (*n* = 317)	50.8% (161)	30.3% (96)	18.9% (60)
Insomnia or excessive need for sleep (*n* = 333)	27.6% (92)	41.1% (137)	31.3% (104)
Use of alcohol/drugs because of event (*n* = 340)	81.5% (277)	14.1% (48)	4.4% (15)
Feelings of shame (*n* = 339)	72.6% (246)	18.9% (64)	8.5% (29)
Feelings of guilt (*n* = 341)	56.6% (193)	30.2% (103)	13.2% (45)
Self-doubts (*n* = 341)	37.2% (127)	39.0% (133)	23.8% (81)
Social isolation (*n* = 339)	68.2% (231)	20.9% (71)	10.9% (37)
Anger towards others (*n* = 336)	58.9% (198)	25.6% (86)	15.5% (52)
Anger towards myself (*n* = 335)	66.8% (224)	23.9% (80)	9.3% (31)
Desire for support by others (*n* = 316)	31.0% (98)	47.8% (151)	21.2% (67)
Desire to process the event for better understanding (*n* = 315)	22.2% (70)	34.9% (110)	42.9% (135)

Most incidents leading to SVP were not related to the COVID-19 pandemic (90.7%, *n* = 313)

All support measures suggested to the participants were rated as helpful. The most important support measures were the possibility of accessing legal consultation after a severe event as well as access to professional counseling or psychological/psychiatric consultations. Table 4 shows the results of the ratings of the support measures.

**Table 4:** Ratings of support measures

Support measure	Not helpful (*n*)	Rather not helpful (*n*)	Rather helpful (*n*)	Very helpful (*n*)
The possibility to take time off from work directly to process the event (*n* = 503)	2.40% (12)	11.50% (58)	38.00% (191)	48.10% (242)
Access to professional counselling or psychological/ psychiatric consultations (crisis intervention) (*n* = 510)	0.80% (4)	2.70% (14)	25.50% (130)	71.00% (362)
The possibility to discuss my emotional/ethical thoughts (*n* = 509)	1.80% (9)	8.60% (44)	37.90% (193)	51.70% (263)
Clear and timely information regarding the course of action after a serious event (e.g., damage analysis, error report) (*n* = 485)	2.70% (13)	7.60% (37)	38.80% (188)	50.90% (247)
Formal emotional support in the sense of organized collegial help (*n* = 494)	1.20% (6)	8.10% (40)	43.30% (214)	47.40% (234)
Informal emotional support (*n* = 484)	2.30% (11)	18.80% (91)	44.80% (217)	34.10% (165)
Quick processing of the situation/quick crisis intervention (in a team or individually) (*n* = 513)	1.40% (7)	3.50% (18)	27.10% (139)	68.00% (349)
Support/Mentoring when continuing to work with patients (*n* = 457)	3.30% (15)	24.70% (113)	44.90% (205)	27.10% (124)
Support when communicating with patients and/or relatives (*n* = 451)	8.00% (36)	31.90% (144)	36.60% (165)	23.50% (106)
Guidelines regarding the role/activities expected of me during a serious event (*n* = 475)	7.80% (37)	25.30% (120)	40.60% (193)	26.30% (125)
Support to be able to take an active role in the processing of the event (*n* = 484)	1.70% (8)	7.60% (37)	52.90% (256)	37.80% (183)
A secure possibility to give information on how to prevent similar events in the future (*n* = 478)	1.90% (9)	6.50% (31)	44.40% (212)	47.30% (226)
The possibility to access legal consultation after a severe event (*n* = 486)	0.80% (4)	2.30% (11)	21.60% (105)	75.30% (366)

The preferences for potential support measures were analyzed between the two groups, SVs and non-SVs. The Mann‒Whitney U test indicated that there were significant differences regarding the rating of the possibility of taking time off from work directly to process the event (*p* = 0.015, *r*=-0.106) and access to professional counseling of psychological/psychiatric consultations (*p* = 0.022, *r*=-0.100). Both support measures were slightly favored by participants who did not identify as SVs.

### Explanatory variables for identifying as a second victim

Our binary logistic regression model revealed that gender, work experience, and working in ground-based intensive care transportation appeared to be significant predictors of SV development. Specifically, men were 60.9% less likely to report being SVs than women (odds ratio (OR) = 0.391, 95%-CI [0.251,0.609], *p* < 0.001). Every additional year of work experience sees an increase of the prevalence of self-reported SV by 5.5% points (OR = 1.055, 95%-CI [1.029,1.081], *p* < 0.001). Compared with those who did not work in that area, those who did work in ground-based intensive care transportation also had an increased likelihood of becoming an SV (OR = 2.371, 95%-CI [1.287,4.368], *p* = 0.006). Personality trait “conscientiousness” goes along with a significant lower rate of experiencing SVP (OR = 0.776, 95%-CI [0.599,0.980], *p* = 0.034).

Table 5 shows the results of the first binary logistic regression.

**Table 5:** Risk factors associated with becoming SVs according to binary logistic regression (*n* = 526)

Predictor	Regression Coefficient B	*p*	Odds Ratio* (Exponentiation of the B Coefficient (Exp(B))1	Odds Ratio 95% CI Lower	Odds Ratio 95% CI Upper
**Male gender** ^**1**^	**-0.94**	**< 0.001**	**0.391**	**0.251**	**0.609**
**Professional experience (years)**	**0.053**	**< 0.001**	**1.055**	**1.029**	**1.081**
Qualified patient transportation	-0.387	0.052	0.679	0.460	1.003
Air-bound rescue	3.398	0.058	29.916	0.895	999.834
**Ground-based intensive care transportation**	**0.863**	**0.006**	**2.371**	**1.287**	**4.368**
Air-bound intensive care transportation	-2.317	0.119	0.099	0.005	1.816
Neonatal ambulance	0.506	0.264	1.658	0.683	4.027
Heavy weight transportation	-0.435	0.140	0.647	0.363	1.153
Advanced rescue services	0.323	0.157	1.382	0.883	2.163
Extraversion	-0.063	0.535	0.939	0.770	1.146
Agreeableness	0.018	0.879	1.019	0.804	1.291
**Conscientiousness**	**-0.267**	**0.034**	**0.776**	**0.599**	**0.980**
Neuroticism	0.030	0.792	1.031	0.823	1.292
Openness	0.033	0.743	1.034	0.847	1.261

^1^referent category is female. **Bold** = significant. Nagelkerke´s pseudo-R²=0.138, AUC = 0.678

*Factors influencing the symptom load of SVs*

Gender was a significant factor influencing the symptom load of the SVs. Persons identified as male were 44.9% less likely to experience a high symptom load than women were (*p* = 0.027). Additionally, persons with higher values for the extraversion were less likely to experience a higher symptom load (*p* = 0.013, Table 6).

**Table 6:** Factors influencing overall symptom load (high vs. low) according to binary logistic regression (*n* = 344)

Predictor	Regression Coefficient B	*p*	Odds Ratio* (Exponentiation of the B Coefficient (Exp(B))1	Odds Ratio 95% CI Lower	Odds Ratio 95% CI Upper
**Male gender** ^**1**^	**-0.596**	**0.027**	**0.551**	**0.325**	**0.934**
Professional experience (years)	0.012	0.371	1.012	0.986	1.038
Event without patient harm² (near miss)	0.149	0.774	1.161	0.420	3.210
Unexpected death/suicide of a patient²	-0.668	0.073	0.513	0.247	1.064
Unexpected death/suicide of a colleague²	-0.119	0.797	0.888	0.359	2.199
Aggressive behavior of patients/relatives²	-0.382	0.354	0.683	0.305	1.530
Other type of key event²	-0.537	0.246	0.584	0.236	1.449
Support received³	-0.206	0.398	0.814	0.505	1.312
**Extraversion**	**-0.283**	**0.013**	**0.754**	**0.603**	**0.941**
Agreeableness	-0.073	0.593	0.930	0.713	1.213
Conscientiousness	-0.103	0.470	0.902	0.682	1.193
Neuroticism	0.034	0.782	1.035	0.811	1.320
Openness	0.192	0.098	1.211	0.965	1.520

^1^as opposed to female gender, ^2^as opposed to an event with patient harm, ^3^as opposed to no support. **bold** = significant. Nagelkerke’s pseudo-R²=0.094, AUC = 0.653

## Discussion

The study revealed a high prevalence of SVP among EMTs, with 65.3% identifying as second victims after learning about the term. 60.2% of the respondents were male, compared to 66.0% within the German EMT population consisting of 86.000 employees, while valid data on age and years in the profession is not available for this group [23]. Notably, 53.3% reported experiencing SVP within the past 12 months, indicating its recurrence and ongoing impact. The primary events triggering SVP were unexpected patient death (37.1%) and aggressive behavior from patients or their relatives (19.1%). Additional free-text responses underscored the severity of trauma, including cases involving child fatalities and multiple casualties. After facing these key events, most relied on their colleagues for support. The most prevalent symptom of SV in this study was insomnia and the situation was relieved in similar professional contexts. With respect to the risk factors for becoming SVs, we found that work experience and working in ground-based intensive care transportation increased the risk of experiencing SVP. However, male gender and elevated levels of personality trait conscientiousness have been shown to decrease the likelihood of becoming SVs. With respect to the overall symptom load after experiencing SVP, we identified male gender and higher levels of extraversion as protective factors. Regarding support measures, access to legal consultation and professional counseling were rated most helpful by the EMTs of this study. Furthermore, non-SVs favor the possibility of taking time off from work and access to professional counseling as support measures compared with EMTs who have already experienced SVP. The prevalence reported in this study is slightly higher than the prevalence described among other health care workers in Germany [9, 10, 12]. EMTs work in an environment where they frequently face chronic and acute stress [24]. Many EMT deployments are associated with high levels of stress due to the unclear situation on site until they arrive [25]. Unexpected adverse events during these deployments, in addition to regular chronic and acute stress, might make EMTs more prone to experiencing SVP and therefore explain the slightly higher prevalence of SVP among the German EMTs in this study than among other medical professionals in Germany [9, 10, 12]. Aggressive behavior of patients or their relatives was the second most common key event leading to the development of SVP in this study. The growing concern over violence against emergency service workers has been highlighted in previous research [26, 27]. A study group from Chile reported that EMTs had a greater risk of encountering violent individuals [28]. A more recent study reported that verbal and physical violence is part of the EMT’s daily work experience in Germany [29]. These findings align with our study’s results, underscoring the significant role such incidents play in the onset of SVP.

Regarding risk factors for experiencing SVP, work experience and working in ground-based intensive care transportation increased the risk. The connection between greater work experience and becoming a SV has already been identified in other SeViD-studies [9, 10]. The current literature also suggests that with increased work experience, the risk of experiencing adverse events increases among other health care workers [30–32]. Ground-based intensive care transportation involves caring for more critically ill patients on average, potentially leading to a higher risk of adverse patient events [33, 34]. The incidence of adverse events during the transportation of critically ill patients is estimated to be between 1.7% and 75.7% [35]. This could explain our finding that EMTs working in these specific areas face a greater risk of developing SVP.

Conversely, we found that male gender and higher levels of conscientiousness may serve as protective factors against the self-reported development of being a second victim. Male gender was identified as a potential protective factor in two previous SeViD-studies [10, 36]. The difference in SVP risk between genders may be shaped by distinct work environments and roles predominantly held by men and women, indicating that both job-related factors and gender-specific personality traits play key roles in vulnerability to SVP [37, 38]. Factors such as variations in emotional labor and coping strategies could further contribute to this disparity, although additional research is needed to fully grasp the extent and nature of these influences. In contrast, women’s greater willingness to openly discuss mental health issues compared to men could have a protective effect by facilitating identification and support [39]. Given the high heterogeneity among individuals, a holistic approach is essential to ensure effective support and prevention strategies for all healthcare providers, regardless of gender or other differences.

Conscientiousness might serve as a protective factor against reporting SVP for two underlying reasons. First, conscientiousness is linked to higher levels of resilience, meaning that individuals who score high in conscientiousness tend to also exhibit greater resilience. Resilience, in turn, is associated with better mental health outcomes and effective coping strategies, possibly mitigating the impact of stress and adverse events [40, 41]. Second, conscientiousness is associated with improved emotional regulation, particularly in recovering more effectively from negative emotional stimuli [42]. This implies that individuals with greater conscientiousness may be better prepared to manage emotional stress, thereby lowering the risk of adverse emotional outcomes such as SVP. Further, we discovered that EMTs in this study who scored higher in extraversion, exhibited a lower overall symptom load. Extraversion is, as conscientiousness is as well, linked to higher levels of resilience since it may enhance the ability to maintain mental health after adverse events [43]. Moreover, extraverted individuals recognize and engage with social support more effectively, benefiting from both its availability and active use [44, 45]. Extraversion is also linked to increased social engagement and a greater tendency to seek support in stressful situations, fostering more effective coping mechanisms [45, 46]. Social support, particularly from peers, has been shown to be an effective mitigator of the effects of SVP [47]. This is further supported by peer support being suggested as an early and low-threshold support measure for SVs [8].

### Limitations

This explorative study has several limitations. First, the cross-sectional design limits our ability to determine causal relationships between the identified factors and SVP. Second, the use of a convenience sample may introduce selection bias, potentially leading to an overestimation of the effect. However, the prevalence of SVP in our study aligns with that reported in previous SeViD studies, indicating that our findings are consistent with external evidence. Another possible limitation is social desirability bias, which might have influenced the responses of our participants. The EMTs who participated in this study may have been inclined to give responses that they thought were socially acceptable or aligned with professional norms, particularly when asked about admitting mistakes or the severity of their symptoms. This bias could result in underreporting of more sensitive topics, such as the impact of SVP on mental health. Furthermore, symptom load was assessed only by participants who identified themselves as Second Victims. Prior findings from the SeViD-IX study revealed that even individuals who did not classify themselves as Second Victims reported SV-like symptoms, likely due to exposure to stressful events [48]. This suggests the presence of “hidden” or “silent” SVs in our sample, which may have resulted in an underestimation of the overall impact of SVP.

Regarding these limitations and the model fit, further refinement and confirmatory tests in large independent samples are needed to clarify the roles of different risk parameters as was shown in the secondary analysis. However, the primary question of the existence and prevalence of SVP in EMT / paramedics could be answered sufficiently and aligns with prior studies. The generalizability of our results to the general experience of EMS personnel is unclear and we encourage researchers from other areas of the world to investigate the prevalence of the SVP within their systems.

## Conclusions

In conclusion, this study provides valuable insights into the prevalence and impact of SVP among EMTs, revealing a high prevalence of 65.3%, with 53.3% experiencing SVP within the past year. The study underscores the significant emotional and psychological toll faced by EMTs, particularly in response to traumatic events such as unexpected patient deaths and violent encounters with patients or their families. The findings suggest that SVP is a recurring issue within this profession, with most EMTs relying on colleagues for support following these events.

Our results highlight work experience and involvement in ground-based intensive care transportation as key risk factors for developing SVP. Male gender and higher levels of conscientiousness showed to have an impact on the question regarding the second victim status with need for clarification. Moreover, the study revealed that higher levels of extraversion were associated with a lower overall symptom load, possibly because these individuals seek social support more effectively. Aside these detailed findings, the high prevalence emphasizes the importance of prevention and social support programs irrespective of any differences in gender or psychological traits. These factors point to the need for tailored support measures, such as the implementation of low-threshold and broadly available peer support programs, to help mitigate the effects of SVP on EMTs. A recently published systematic review confirmed that occupational stress and burnout are highly prevalent among EMS personnel. The authors stated that, given the severity of symptoms, there is a lack of support for EMS employees and that designing and implementing support programs was mandatory to reduce the negative consequences [49]. Becoming a second victim may lead to dysfunctional coping strategies, including changes in work behavior and practicing defensive medicine. This may result in either undertreatment or overtreatment, both threatening patient safety [50]. Together with researchers from Austria, members of our study group have developed a low-threshold concept for collegial help in an Austrian hospital [51]. There is some evidence that peer support may result in major savings in health expenses and recruiting [52]. To this date, peer support systems are widely lacking within the German EMS environment. Establishing these systems is not merely an ethical issue but has been shown to have a major impact on employee health and intention to leave the job or even the profession, at least as far as other professional groups as nurses and physicians are concerned. To identify the economic impact of peer support on German EMS personnel, we are currently performing a simulation study using a Markov model [53].

Overall, the findings highlight the need for increased awareness, implementation of support systems and preventive measures to better address SVP in EMTs, ultimately improving their well-being and professional performance.

## Data Availability

The data presented in this study are available upon request from the corresponding author.

## Abbreviations

AUC: Area under the curve
BFI: Big Five Inventory
CI: Confidence interval
EMS: Emergency medical service
EMT: Emergency medical technician
M: Mean
OR: Odds ratio
ROC: Receiver operating characteristic
SD: Standard deviation
SeViD: Second Victims in German-speaking Countries
SV: Second Victim
SVP: Second Victim Phenomenon
VIF: Variance Inflation Factor
